# Prenatal Diagnosis of Central Nervous System Anomalies by High-Resolution Chromosomal Microarray Analysis

**DOI:** 10.1155/2015/426379

**Published:** 2015-05-12

**Authors:** Lijuan Sun, Qingqing Wu, Shi-Wen Jiang, Yani Yan, Xin Wang, Juan Zhang, Yan Liu, Ling Yao, Yuqing Ma, Li Wang

**Affiliations:** ^1^Department of Ultrasound, Beijing Obstetrics and Gynecology Hospital, Capital Medical University, Beijing 100026, China; ^2^Department of Biomedical Science, Mercer University School of Medicine, Department of Obstetrics and Gynecology, Memorial Health Hospital, 4700 Waters Avenue, Savannah, GA 31404-3089, USA; ^3^Department of Obstetrics, Beijing Obstetrics and Gynecology Hospital, Capital Medical University, Beijing 100026, China

## Abstract

The aims of this study were to evaluate the contribution of chromosomal microarray analysis (CMA) in the prenatal diagnosis of fetuses with central nervous system (CNS) anomalies but normal chromosomal karyotype. A total of 46 fetuses with CNS anomalies with or without other ultrasound anomalies but normal karyotypes were evaluated by array-based comparative genomic hybridisation (aCGH) or single-nucleotide polymorphism (SNP) array. The result showed that CNVs were detected in 17 (37.0%) fetuses. Of these, CNVs identified in 5 (5/46, 10.9%) fetuses were considered to be likely pathogenic, and CNVs detected in 3 (3/46, 6.5%) fetuses were defined as being of uncertain clinical significance. Fetuses with CNS malformations plus other ultrasound anomalies had a higher rate of pathogenic CNVs than those with isolated CNS anomalies (13.6% versus 8.3%), but there was no significant difference (Fisher's exact test, *P* > 0.05). Pathogenic CNVs were detected most frequently in fetuses with Dandy-Walker syndrome (2/6, 33.3%) when compared with other types of neural malformations, and holoprosencephaly (2/7, 28.6%) ranked the second. CMA is valuable in prenatal genetic diagnosis of fetuses with CNS anomalies. It should be considered as part of prenatal diagnosis in fetuses with CNS malformations and normal karyotypes.

## 1. Introduction

The prevalence of CNS abnormalities is 0.14–0.16% in live births and reaches as high as 3–6% in stillbirths [1]. CNS anomalies are a group of serious birth defects associated with high rates of infant death or disability. In addition to their threat to life, CNS malformations cause enormous direct and indirect health costs [2]. While the etiology of fetal central nervous system anomalies is highly heterogeneous, genetic conditions are recognized as a major cause [3, 4]. The significance of genetic mutations is also underscored by the fact that many environmental factors lead to CNS malformations through their mutagenic effects. Studies have shown that CNS malformations detected by ultrasonography were strongly associated with chromosomal abnormalities, especially trisomy 13 and 18 [5, 6]. However, there remains a dilemma in prenatal diagnosis of fetuses who have CNS anomalies, either with or without other organ abnormalities, but have normal karyotypes.

Conventional chromosome karyotype analysis such as G-Banding has been the standard method for the detection of a wide range of chromosomal abnormalities in the past decades. However, this technique is limited to the detection of chromosomal alterations larger than 5 Mb, and submicroscopic duplications and deletions, which are often associated with mental retardation (MR) and malformations, are not detectable by conventional karyotyping [7].

Chromosomal microarray analysis (CMA) allows the detection of microdeletions and microduplications that are not routinely seen on karyotyping [8]. It is possible to evaluate the entire genome for DNA CNVs, as small as 50–100 kb, which is equal to a 100-fold magnification in resolution compared with karyotyping [9, 10]. This high-resolution analysis of DNA CNVs, initially applied to cancer studies, has subsequently been extended to postnatal diagnosis of various congenital anomalies and MR. Recent studies have focused specifically on the use of microarray analysis in prenatal diagnosis of fetuses with abnormal ultrasound findings [8, 11, 12], especially on the concerns regarding the relationship between CNVs and congenital heart diseases (CHD) [13, 14]. Application of CMA to identify submicroscopic chromosomal aberrations in fetuses with CNS anomalies has been poorly described in the literature so far.

The aims of this study were to evaluate the utility of CMA for prenatal diagnosis of fetuses with CNS anomalies detected by ultrasound but normal karyotypes and to explore the CNVs in fetuses carrying different types of CNS malformations.

## 2. Materials and Methods

### 2.1. Subject Selection and Ultrasound Findings

From December 2011 to June 2014, 31,802 pregnant women were referred to the Department of Obstetrics and Fetal-Maternal Medicine, Beijing Obstetrics and Gynecology Hospital, Capital Medical University, China, for routine anomaly scan. 46 fetuses were diagnosed for CNS abnormalities, with or without other associated anomalies by transabdominal ultrasonography, showing normal karyotypes in conventional G-band karyotype analysis. Of these 46 primary study subjects, in 24 the CNS anomalies were isolated and 22 fetuses with CNS malformations were associated with other abnormalities. This study was approved by the local Ethics Committee of the Capital Medical University. Written informed consent to participate in the study was obtained from each patient.

### 2.2. CMA Methods

Umbilical cord blood samples were collected from the 46 pregnant women between 21 and 27 gestational weeks by cordocentesis. Of the 46 samples, 30 cases (65.2%) were examined with array-based comparative genomic hybridisation (aCGH), and then additional 16 cases (34.8%) were tested with single-nucleotide polymorphism (SNP) array in consideration of the advantages for SNP detecting copy number aberrations [15] after being approved by Food and Drug Administration (FDA). Genomic DNA was extracted from 2 mL of umbilical cord blood with a commercially available Blood Genomic DNA Extraction kit (BioChain Institute Inc., Newark, CA or Yuanpinghao Biotech Co., Ltd., resp.) according to the manufacturer's instructions. aCGH was performed using 8 × 60 K oligonucleotide-based microarray (Agilent), and SNP array was detected using a 750 K microarray (Affymetrix CytoScan 750 K Array). After hybridization, a laser scanner was used for scanning the arrays, and the data was analyzed with the use of special software package (Workbench and Chromosome Analysis Suite).

### 2.3. The Interpretation for CMA Results

To interpret the results, we compared all detected copy number gains or losses with known CNVs listed in publically available databases (DECIPHER Database (http://decipher.sanger.ac.uk/), Online Mendelian Inheritance in Man (OMIM, http://omim.org/), PubMed (http://www.ncbi.nlm.nih.gov/pubmed/), and the Database of Genomic Variants (DGV, http://www.ncbi.nlm.nih.gov/dbvar/). For all CNVs, CMA results were interpreted independently of any previous cytogenetic findings.

### 2.4. Statistical Analysis

Statistical analysis of data was performed using SPSS version 17.0. Bivariate analysis was carried out using Fisher's exact test (two-tailed). *P* < 0.05 was considered statistically significant.

## 3. Results 

### 3.1. CNVs Detection Rates

Of the 46 study subjects, CNVs were detected in 17 (37.0%) cases, whereas no deletion or duplication was found in the remaining 29 cases (63.0%). Pathogenic CNVs were identified in 5 (10.9%) fetuses including 2 cases with isolated CNS malformations and 3 cases associated with other organ abnormalities. In addition, CNVs of uncertain clinical significance were detected in 3 (6.5%) fetuses, and CNVs identified in 9 (19.6%) fetuses were considered to be likely benign and of no clinical significance. Fetuses with CNS malformations plus other ultrasound anomalies had a seemingly higher detection rate than those with isolated CNS anomalies (13.6% versus 8.3%), but the difference did not reach a statistically significant level (Fisher's exact test, *P* > 0.05) (Table 1).

### 3.2. CMA Results for 5 Fetuses with Pathogenic CNVs and 3 Cases with CNVs of Uncertain Clinical Significance

**Figure 1:**
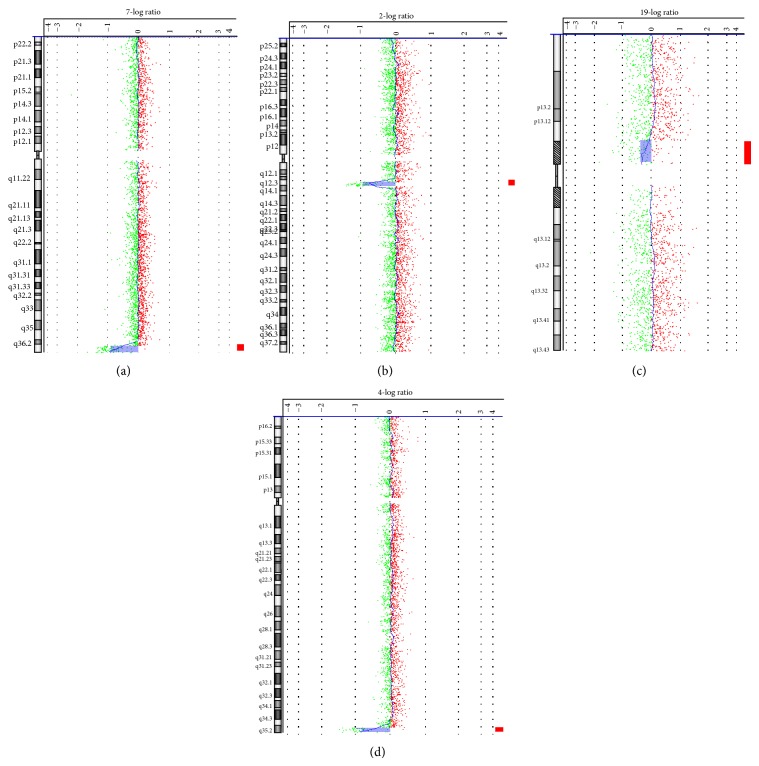
Microarray testing results. The CNVs of Cases 1, 3, 6, and 7 were detected by aCGH. (a)–(d) showed aCGH results of Cases 1, 3, 6, and 7, respectively. (a) A 3.44-Mb deletion within chromosome 7q in Case 1. (b) A 3.25-Mb deletion in chromosome 2q in Case 3 harbored four OMIM genes. (c) A 4.03-Mb deletion of chromosome 19p in Case 6. (d) A 2.79-Mb deletion in chromosome 4q35.2 in Case 7. The respective chromosomes are shown and labeled. Signal intensity is plotted on a log⁡_2_⁡ scale, such that a normal copy number gives a value of 0. Chromosomal deletions are denoted by leftward deviation of the central line (marked by red boxes).

**Figure 2:**
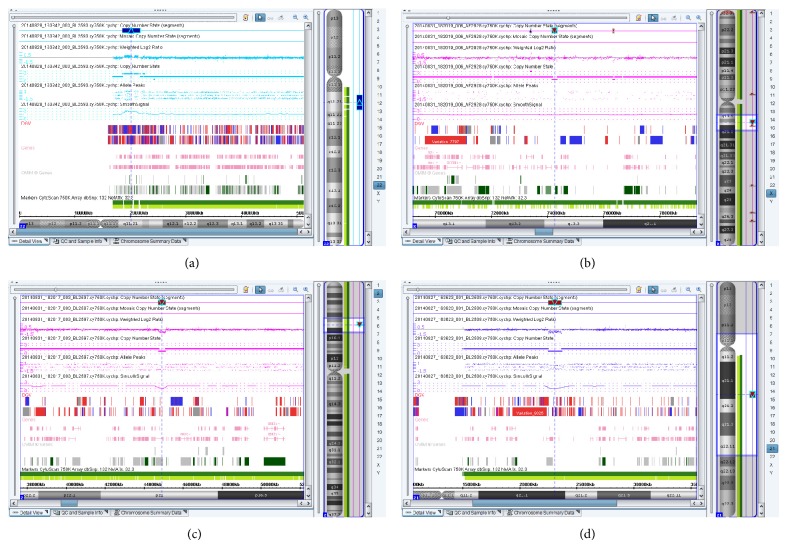
Microarray testing results. The CNVs of Cases 2, 4, 5, and 8 were detected by SNP array. (a)–(d) showed the SNP results of Cases 2, 4, 5, and 8, respectively. (a) A 3.15-Mb duplication in chromosome 22q11.21 included 43 OMIM genes in Case 2. (b) A 0.17-Mb deletion in chromosome Xq13.3 in Case 4. (c) A 0.34-Mb deletion within chromosome 2p21 in Case 5. (d) A 1.15-Mb deletion of chromosome 21q21.1 in Case 8. The chromosome numbers and cytobands are shown and labeled on the right side. The view on the left side shows the detected segments, regions, and reference annotations in detail. Chromosomal duplication segments are denoted by upward triangle (blue) whereas deletion segments are denoted by downward triangle (red).

Information for 5 fetuses with pathogenic CNVs and 3 cases with CNVs of uncertain clinical significance was shown in Figures 1 and 2, and Table 2.

Of the 24 fetuses with isolated CNS anomalies, pathogenic CNVs were identified in 2 cases (2/24, 8.3%) (Table 2, Cases 1 and 2) as follows.

**Figure 3:**
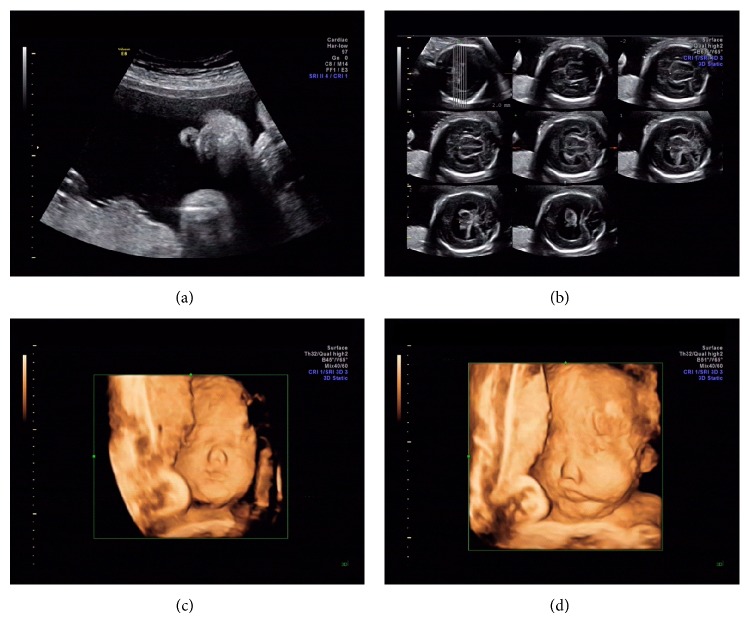
Sonographic findings in Case 1 at 23^+1^ weeks. (a) In two-dimensional coronal plane, single nostril was visualized. (b) Holoprosencephaly was demonstrated in tomographic ultrasound imaging (TUI). ((c) and (d)) Single nostril was further confirmed in three-dimensional imaging.

(1) Case 1 was a fetus with holoprosencephaly and single nostril on prenatal ultrasound. Sonographic abnormal findings were shown in Figure 3 and confirmed by the autopsy pathology. The microarray analysis result showed a 3.44-Mb deletion within the chromosome 7q36.3, encompassing the sonic hedgehog (SHH) gene (Table 2, Figure 1(a)). Deletions SHH gene are known to be related to holoprosencephaly [16] (OMIM ID: ^∗^600725).

(2) Case 2 harbored a 3.15-Mb duplication in chromosome 22q11.21 (Table 2, Figure 2(a)) which included 43 OMIM genes such as TBX1 in a fetus with hydrocephaly. The CMA result has been associated with 22q11.2 microduplication syndrome (OMIM ID: #608363). Dupont et al. reported that 22q11.2 microduplication syndrome was characterized by a highly variable clinical phenotypes, ranging from apparently normal or slightly dysmorphic features to severe malformations with profound mental retardation [17]. Neurological features of the syndrome included intellectual or learning disability, motor delay, and other neurodevelopmental disorders [18]. In this case, the parents chose termination of pregnancy (TOP) because of fetal hydrocephaly that was confirmed by postnatal imaging.

Information for clinically significant CNVs detected in 3/22 (13.6%) fetuses with CNS anomalies as well as other abnormalities (Table 2, Cases 3–5) was as follows.

(1) Case 3 was a fetus with Dandy-Walker malformation in association with ventricular septal defect and persistent left superior vena cava. The microarray test result revealed a 3.25-Mb deletion in chromosome 2q13–q14.1 that included four OMIM genes (Table 2, Figure 1(b)): PAX8, IL1B, MERTK, and IL1RN. As reported by Kasai and Narahara, these genes were associated with neurodevelopmental impairment, congenital heart defects, and facial and finger malformations [19]. The parents chose TOP because of the sonographic abnormalities that were confirmed by autopsy.

(2) Case 4 carried a 0.17-Mb deletion in chromosome Xq13.3 (Table 2, Figure 2(b)) which involved the ABC7 gene (OMIM ID: ^∗^300135) in a male fetus with Dandy-Walker syndrome (cerebellar malformation), absence of septum pellucidum, and arachnoid cyst associated with lip and palate cleft and skeletal dysplasia. Bekri et al. [20] previously identified a missense mutation in the ABC7 gene to be the cause of X-linked sideroblastic anemia with cerebellar ataxia. Although it is not experimentally confirmed, we considered that the microdeletion might be responsible for fetal cerebellar malformation. The parents opted for TOP because of abnormal sonographic findings which were confirmed by autopsy pathology.

(3) Case 5 was a fetus with holoprosencephaly (HPE) associated with lip and palate cleft. The microarray test discovered a 0.34-Mb deletion within 2p21 (Table 2, Figure 2(c)) which harbors SIX3 gene (OMIM ID: ^∗^603714). It has been reported that molecular evaluation of foetuses with holoprosencephaly showed high incidence of microdeletions in four HPE genes, one of which was SIX3 gene [21]. Lacbawan et al. reported that SIX3 mutations could result in relatively severe holoprosencephaly [22]. The parents had chosen TOP because of the severe malformations detected by prenatal ultrasound which were confirmed by autopsy.

Our study covered 3 fetuses with the CNVs of uncertain clinical significance (Table 2, Cases 6–8) as the follows.

(1) Case 6 was originally referred for prenatal cytogenetic diagnosis because of exencephaly detected by ultrasonography. Following negative findings in fetal karyotype analysis, CMA result revealed a deletion of 4.03 Mb in chromosome 19p12p13.11 (Table 2, Figure 1(c)). The region contained a lot of segmental duplications and many homologous genes, such as ZNF family that probably mediated the recombination giving rise to the deletion. However, extensive literature search failed to identify that any gene in the relevant region might cause known syndromes. Both parents showed negative CMA results, indicating that the deletion was de novo. Thus, the clinical significance of this CNV was uncertain. The family opted for TOP because of the severe ultrasonography results. The patient underwent Rivanol amniocentesis induction of labor with an informed consent, and the autopsy pathology confirmed the ultrasound diagnosis.

(2) Case 7 was referred because of holoprosencephaly in association with facial anomaly and ventricular septal defect as ultrasonographic findings. There was a 2.79-Mb deletion in chromosome 4q35.2 (Table 2, Figure 1(d)), with proximal breakpoint located 300 kb upstream the OMIM gene FAT1. This gene encodes a tumor suppressor essential for controlling cell proliferation during* Drosophila* development [23]. The gene product is a member of the cadherin superfamily and is expressed at high levels in a number of fetal epithelia. Its product probably functions as an adhesion molecule and/or signaling receptor and is likely to be important in developmental processes and cell communication. We think that it is possible for the microdeletion to influence FAT1 gene expression by position effect because of the near distance. The CMA results from both of parents were negative, indicating a de novo mutation. So the clinical significance of the CNVs in this case remained unclear. The parents chose TOP, and the abnormal ultrasound findings were confirmed by autopsy pathology.

(3) Case 8 was a fetus with hydrocephaly associated with sacrococcygeal vertebral anomaly, intrauterine growth retardation (IUGR), and thickened nuchal fold (NF) on prenatal ultrasound. The array test result showed a 1.15-Mb deletion in chromosome 21q21.1 (Table 2, Figure 2(d)) which contained most of the NCAM2 gene. It has been reported that NCAM2 was a candidate for involvement in certain Down syndrome phenotypes [24]. However, the role of NCAM2 deletion in the pathophysiology of Down syndrome is unknown. CMA on both parents was negative, pointing to a possibility for de novo mutation. The clinical significance of this CNV was considered to be uncertain. The pregnant woman underwent TOP, and the autopsy pathology confirmed the ultrasonographic findings.

### 3.3. The Types of Fetal CNS Anomalies and Pathogenic CNVs Incidence

The relationship between the different types of CNS anomalies and the incidence of fetuses with pathogenic CNVs is shown in Table 3. We found that clinically significant CNVs were detected most frequently in fetuses with Dandy-Walker syndrome (2/6, 33.3%), and holoprosencephaly (2/7, 28.6%) ranked the second.

## 4. Discussion 

In the past few years, CMA has been extensively used to investigate chromosomal aberrations in the postnatal population with unexplained neurodevelopmental disorders including developmental delay/intellectual disability, autism spectrum disorders, and multiple congenital anomalies [25–27]. A recent meta-analysis [26] of CMA on 13,926 postnatal subjects in whom conventional cytogenetic tests have proven negative reported an overall diagnostic rate of 10% for pathogenic genomic imbalances in such populations.

Recently results on application of CMA for prenatal diagnosis have also been published. D'Amours et al. [12] reported a 8.2% detection rate of pathogenic CNVs in fetuses with major malformations and a normal karyotype by aCGH. A meta-analysis conducted by Hillman et al. [28] indicated that CMA had a 5.2% additive value compared with conventional karyotyping. Collectively, these studies suggested that CMA was able to identify additional, clinically significant cytogenetic information in fetuses with various kinds of structural anomalies and a normal karyotype. The efforts to diagnose fetuses with CNS malformations with or without other structural abnormalities in our study represent a further attempt for the usage of this powerful technique in prenatal genetic tests.

In our study we focused on fetuses with CNS malformations and an apparently normal karyotype, and the results showed that 10.9% of fetuses had submicroscopic chromosomal abnormalities which were likely to be pathological. This suggests that the microarray technique is able to provide additional information in fetuses with CNS malformations and a normal karyotype. Such information could lead to better prenatal consultation and the assessment of the recurrent risk. Compared with the studies of D'Amours et al. [12] and Hillman et al. [28], this project achieved a higher detection rate (10.9%) of pathogenic CNVs. The elevated detection rate could be attributed to the exclusion of congenital non-CNS anomalies in this study. It should be pointed out that the sample size of this study was relatively small, which could limit the extrapolation of our observations. Further investigations in larger cohorts need to be conducted to validate the potentially benefits of CMA.

In this study we found that the detection rates of pathogenic CNVs in various types of CNS anomalies were different. It was noteworthy that fetuses with Dandy-walker syndrome (2/6, 33%) emerged to be most frequently associated with submicroscopic chromosomal abnormalities among various congenital neural abnormalities when karyotyping showed normal results. There were a few reports [29, 30] regarding CNVs of Dandy-Walker malformation, such as deletion in chromosomes 13q and 7p, which were different from deletion in chromosomes 2q and Xq in this study. Our results also indicated holoprosencephaly (2/7, 28.6%) as the second common malformation associated with pathogenic CNVs among all types of CNS abnormalities. This observation provides some insights into the pathogenesis of holoprosencephaly in fetuses with a normal karyotype, suggesting that CNVs could be a significant cause of this specific type of CNS abnormalities. Shaffer et al. [31] performed a retrospective analysis of pathogenic CNVs detection rate by CMA for 2858 pregnancies with different organ system abnormalities and normal karyotypes and found that both of posterior fossa defects (including Dandy-Walker syndrome and cerebellar hypoplasia) and holoprosencephaly had the highest detection rates of clinically significant CNVs (14.6% and 10.6%) among various types of CNS anomalies, which was similar to our results.

Clinically significant CNVs were detected in 3 fetuses with nervous system anomalies plus other congenital structural malformations, one case with cardiovascular defect, one with lip and palate cleft, and another one with skeletal dysplasia and lip and palate cleft. Fetuses with CNS malformations plus other ultrasound anomalies had a higher detection rate than those with isolated CNS anomalies (13.6% versus 8.3%), but there were no significant differences in the incidence of pathogenic CNVs between them (Fisher's exact test, *P* > 0.05). Our results are comparable with the study reported by Shaffer et al. [31], who found the detection rate of clinical significant CNVs was 6.5% in fetuses with a single CNS anomaly while 11% in cases with CNS malformations and other abnormalities.

Although chromosomal microarray techniques could offer several advantages including high-resolution, whole genome analysis, and a short turnaround time (about 48 h after DNA extraction) in comparison with conventional chromosomal karyotyping, it cannot identify balanced translocations or low level of mosaicism. Therefore, prenatal consultations should not rely on the results of CMA alone, and conventional chromosomal karyotyping and prenatal ultrasound diagnosis should also be required. The present results substantiated that CMA may be especially valuable in routine prenatal diagnosis when fetuses with abnormal ultrasound findings but normal karyotypes.

## 5. Conclusion 

Our results have shown that CMA is a valuable diagnostic tool in prenatal genetic diagnosis of CNS anomalies. Our data indicated that assessment of submicroscopic chromosomal aberrations by CMA should be undertaken in fetuses with CNS anomalies and a normal karyotype. This finding not only provides information for clinical consultation but may also allow more accurate genetic diagnosis and a better understanding of the etiology and mechanisms involved in the congenital defects.

## Figures and Tables

**Table 1:** The detection rates of pathogenic CNVs in fetuses with CNS anomalies associated with different ultrasound findings.

Ultrasound findings	Number of fetuses	Number of fetuses with pathogenic CNVs	Detection rate (%)
Isolated CNS anomaly	24	2	8.3%
Associated with other structural	22	3	13.6%
malformations			
Cardiovascular system	10^#^	1	
Urinary system	2	0	
Musculoskeletal system	6	1^∗^	
Digestive system	1	0	
Tumors	2	0	
Facial anomaly/lip and palate cleft	1	1	

Total	46	5	10.9%

^#^One (Case 7) of 10 fetuses with cardiovascular abnormalities was also associated with facial anomaly, but only in the row of cardiovascular system.

^∗^The fetus (Case 4) was associated with skeletal dysplasia and lip and palate cleft, but only in the row of musculoskeletal system.

**Table 2:** Pathogenic CNVs and variants of unknown significance detected by chromosomal microarray analysis (CMA) in fetuses with CNS anomalies.

Case number	GA	CNS anomalies	Associated anomalies	CMA result	Size (Mb)	CNV type	OMIM or corresponding disorder
1	23+	Holoprosencephaly, single nostril	No	arr7q36. (155,473,296–158,909,738) × 1	3.44	Loss	SHH gene (^∗^600725)

2	22+	Hydrocephaly	No	arr22q11.21 (18,648,855–21,800,471) × 3	3.15	Gain	22q11.2 duplication syndrome (^#^608363)

3	25+	Dandy-Walker syndrome	VSD PLSVC	arr2q13q14.1 (111,596,906–114,844,660) × 1	3.25	Loss	PAX8 (^∗^167415) IL1B (^∗^147720) MERTK (^∗^604705) IL1RN (^∗^147679)

4	23+	Dandy-Walker syndrome	Skeletal dysplasia lip and palate cleft absence of septum pellucidum arachnoid cyst	arrXq13.3 (74,171,888–74,343,340) × 1	0.17	Loss	ABC7 (^∗^300135)

5	23+	Holoprosencephaly	Lip and palate cleft	arr2p21 (44,749,075–45,098,283) × 1	0.34	Loss	SIX 3 gene (^∗^603714)

6	21+	Exencephaly	No	arr19p12p13.11 (19,838,485–23,868,512) × 1	4.03	Loss	

7	24+	Holoprosencephaly	VSD facial anomaly	arr4q35.2 (187,979,723–190,767,114) × 1	2.79	Loss	

8	25+	Hydrocephaly	Thickened NF IUGR sacrococcygeal vertebral anomaly	arr 21q21.1 (22,508,434–23,663,338) × 1	1.15	Loss	

GA, gestational weeks; VSD, ventricular septal defect; PLSVC, persistent left superior vena cava; NF, nuchal fold; IUGR, intrauterine growth retardation.

Human genome build was hg19.

**Table 3:** The types of fetal CNS anomalies and pathogenic CNVs incidence.

CNS anomalies classification	Number of fetuses	Number of fetuses with pathogenic CNVs
Anencephaly	1	0
Exencephaly	1	0
Dandy-Walker syndrome	6	2 (33.3%)
Holoprosencephaly	7	2 (28.6%)
Spinal bifida	9	0
Intracranial tumor (ICT)	1	0
Hydrocephaly	8	1 (12.5%)
Schizencephaly	1	0
Agenesis of the corpus callosum (ACC)	2	0
Choroid plexus cyst	2	0
Arachnoid cyst	2	0
Cerebellar hypoplasia	1	0
Subependymal cyst	1	0
Encephalocele/meningoceles	1	0
Other CNS malformation	3	0

Total	46	5 (10.9%)
